# Transcriptomic Analysis of *Paeonia delavayi* Wild Population Flowers to Identify Differentially Expressed Genes Involved in Purple-Red and Yellow Petal Pigmentation

**DOI:** 10.1371/journal.pone.0135038

**Published:** 2015-08-12

**Authors:** Qianqian Shi, Lin Zhou, Yan Wang, Kui Li, Baoqiang Zheng, Kun Miao

**Affiliations:** State Key Laboratory of Tree Genetics and Breeding, Key Laboratory of Tree Breeding and Cultivation of State Forestry Administration, Research Institute of Forestry, Chinese Academy of Forestry, Beijing 100091, China; Nazarbayev University, KAZAKHSTAN

## Abstract

Tree peony (*Paeonia suffruticosa* Andrews) is a very famous traditional ornamental plant in China. *P*. *delavayi* is a species endemic to Southwest China that has aroused great interest from researchers as a precious genetic resource for flower color breeding. However, the current understanding of the molecular mechanisms of flower pigmentation in this plant is limited, hindering the genetic engineering of novel flower color in tree peonies. In this study, we conducted a large-scale transcriptome analysis based on Illumina HiSeq sequencing of cDNA libraries generated from yellow and purple-red *P*. *delavayi* petals. A total of 90,202 unigenes were obtained by *de novo* assembly, with an average length of 721 nt. Using Blastx, 44,811 unigenes (49.68%) were found to have significant similarity to accessions in the NR, NT, and Swiss-Prot databases. We also examined COG, GO and KEGG annotations to better understand the functions of these unigenes. Further analysis of the two digital transcriptomes revealed that 6,855 unigenes were differentially expressed between yellow and purple-red flower petals, with 3,430 up-regulated and 3,425 down-regulated. According to the RNA-Seq data and qRT-PCR analysis, we proposed that four up-regulated key structural genes, including *F3H*, *DFR*, *ANS* and *3GT*, might play an important role in purple-red petal pigmentation, while high co-expression of *THC2'GT*, *CHI* and *FNS II* ensures the accumulation of pigments contributing to the yellow color. We also found 50 differentially expressed transcription factors that might be involved in flavonoid biosynthesis. This study is the first to report genetic information for *P*. *delavayi*. The large number of gene sequences produced by transcriptome sequencing and the candidate genes identified using pathway mapping and expression profiles will provide a valuable resource for future association studies aimed at better understanding the molecular mechanisms underlying flower pigmentation in tree peonies.

## Background

Tree peony belongs to section *Moutan* of the genus *Paeonia*, family Paeoniaceae, is an important traditional ornamental and medicinal plant in China, and has been named ‘the king of flowers’ for its large, showy and colorful flowers [1]. There are nine wild species of tree peony, *P*. *suffruticosa*, *P*. *cathayana*, *P*. *jishanensis*, *P*. *qiui*, *P*. *ostii*, *P*. *rockii*, *P*. *decomposita*, *P*. *delavayi* and *P*. *ludlowii* [2–4], and about 1,500 cultivars with a wide range of flower colors that have been produced across the world by conventional breeding. Among these species, *P*. *delavayi* is very special, with extreme variability both within and between populations in the number, length, and width of leaf segments and the number, size, and color of all its floral parts [5]. These plants are distributed mainly in the northwest of Yunnan Province, southwest of Sichuan Province and southeast of Tibet, China [5,6]. Some papers and our previous study have reported various petal colors in the same population of *P*. *delavayi*, including yellow, orange, red, dark red, or purple-red [5,7]. Plants with yellow flowers are considered the most precious resource for cultivar breeding because traditional Chinese cultivar flowers are usually purple, pink, red or white, but lacking in pure yellow. Moreover, Li et al. [8] reported that the antioxidant activity of *P*. *delavayi* was higher than that of other species with yellow flowers, which have been considered potential resources for the development of new drugs or functional foods, demonstrating the great economic significance of research on this species.

Among the three major groups of pigments, flavonoids, particularly anthocyanidins, are the most widely distributed in higher plants and contribute to a wide range of colors, from pale yellow to red, purple and blue [9,10]. To date, more than 30 different flavonoids, including anthocyanins and multiform glycosides of flavones and flavonols, have been identified and quantified from different groups and several wild species of tree peony [11–15]. Wang et al. [12] analyzed the composition and content of flower pigments in seven wild tree peonies and found no pelargonidin (Pg)-based anthocyanins in any accessions in subsection Delavayanae; *P*. *delavayi* with purple flowers mainly contained peonidin-3,5-glucosides (Pn3G5G). In a recent study, Li et al. [8] examined the composition and content of flavonoids from yellow flowers of *P*. *delavayi*, and identified the main compound as chalcone 2′-glucoside (isosalipurposide, ISP). In our previous research [16], we investigated the pigment composition of yellow petals from a wild *P*. *delavayi* population in Yunnan Province, China, and found that chalcones, flavones and flavonols were the main components, including ISP, kaempferol, quercetin, isorhamnetin, chrysoeriol and apigenin-glycopyranoside [16]. These studies provide a physiological and biochemical basis for future research on the molecular mechanism of *P*. *delavayi* flower pigmentation.

Flavonoids are synthesized through the phenylpropanoid biosynthesis pathway, which can be divided into three stages. The first stage is the conversion of phenylalanine to coumaroyl-CoA, which is shared in many secondary metabolism pathways. The second stage is the synthesis of dihydroflavonols such as dihydrokaempferol (DHK), dihydroquercetin (DHQ) and dihydromyricetin (DHM) from one molecule of coumaroyl-CoA and three molecules of malonyl-CoA catalyzed by a series of enzymes including chalcone synthase (CHS), chalcone isomerase (CHI), flavanone 3-hydroxylase (F3H), flavonoid 3’-hydroxylase (F3’H), flavonoid 3’5’-hydroxylase (F3’5’H), flavone synthase (FNS) and flavonol synthase (FLS). The third stage is the synthesis of various anthocyanidins from dihydroflavonols catalyzed by dihydroflavonol 4-reductase (DFR) and anthocyanidin synthase (ANS), followed by the formation of stable anthocyanins through a series of glycosylation and methylation reactions catalyzed by anthocyanidin 3-*O*-glucosyltransferase (3GT), anthocyanidin 5-*O*-glucosyltransferase (5GT) and anthocyanin *O*-methyltransferase (AOMT) [17,18]. In the flavonoid biosynthetic pathway, the transcription levels of flavonoid biosynthesis genes are regulated by various transcription factors, which can be classified into three families: R2R3-MYB, basic-Helix-Loop-Helix (bHLH) and WD40 [19]. To date, a large number of structural genes as well as some regulatory genes have been well characterized and isolated in mutants or crossed lines of snapdragon, maize, petunia and *Arabidopsis* as model plants [17,18]. Meanwhile, numerous studies on the molecular mechanism of pigmentation have been performed in different ornamental plants, including *P*. *suffruticosa*, in which five structural genes (*PsCHS1*, *PsCHI1*, *PsANS1*, *PsF3H1* and *PsDFR1*) were cloned and identified as the most important genes involved in tree peony flower pigmentation [20–22]. However, despite the economic and breeding importance of *P*. *delavayi*, flower pigmentation in this plant has previously been studied only at the physiological level [8,16]. Therefore, studying the molecular biology of *P*. *delavayi* color formation is of great importance to accelerate the use of the unique flower genes in this species.

For woody plants, especially those of high heterozygosity, such as tree peony, whole genome sequencing requires long-term and expensive investment. Transcriptome sequencing based on the Illumina/Solexa high-throughput sequencing platform is a rapid and convenient way to obtain information on the expressed fraction of genome [23,24]. RNA-Seq is not restricted by a reference sequence and is suitable for non-model organisms without genomic sequences [25–28]. It has been widely applied to model as well as non-model organisms in various studies, including transcript profiling, single nucleotide polymorphism discovery and the identification of genes that are differentially expressed between samples [29–34].

In the present work, individuals with purple-red and yellow flowers within a wild *P*. *delavayi* population in Yunnan Province, China were used as experimental materials, and two transcriptomes (of petals of each color mixed at different flower opening stages) were sequenced on the Illumina HiSeq 2000 platform. Additionally, by analyzing the data with various bioinformatics tools, we discovered some potential differentially expressed genes involved in purple-red and yellow petal pigmentation, and analyzed the expression profiles of seven candidate genes through real-time PCR. To the best of our knowledge, this is the first exploration of the petal transcriptome of the wild tree peony species *P*. *delavayi*. These transcriptome sequences may provide a theoretical basis to understand the molecular mechanisms behind the pigment composition of *P*. *delavayi* and a valuable resource for identifying genes expressed in this wild population.

## Results

### Illumina sequencing and *de novo* transcriptome assembly

To achieve a broad survey of the genes associated with *P*. *delavayi* flower pigmentation, two cDNA libraries (Pl and Pd) were generated for RNA-Seq. Each included a mixture of equal amounts of RNA from petals at five flower opening stages (Pl, yellow petals; Pd, purple-red). The mRNA was isolated, enriched, sheared into smaller fragments, and reverse-transcribed into cDNA, which was subjected to sequencing on an Illumina HiSeq 2000 sequencing platform. In total, we got 52.2 million raw reads with an average length of 83.42 nt and 54.3 million reads with an average length of 84.94 nt from the Pl and Pd sequencing libraries, respectively. After removal of adaptor sequences, ambiguous reads, and low-quality reads, 48,401,848 high-quality clean reads comprising 4,356,166,320 nucleotides (4.35 Gb) with a Q20 percentage of 98.31% and a GC percentage of 45.47% were generated from Pl transcriptome sequencing, while 51,300,426 high-quality clean reads comprising 4,617,038,340 nucleotides (4.61 Gb) with the same Q20 percentage as that of Pl and a GC percentage of 45.69% were generated from Pd transcriptome sequencing (Table 1). All high-quality reads were assembled *de novo* by the Trinity program [35]. From the Pl dataset, 149,470 contigs were produced with an N50 of 542 nt (i.e. 50% of the assembled bases were incorporated into contigs of 542 nt or longer), while 144,205 contigs with an N50 of 579 nt were generated from the Pd sample. After linking the contigs together, we obtained 88,330 unigenes with an average length of 603 nt from the Pl dataset, most of which were 200–3000 bp long, and 82,720 unigenes with an average length of 615 nt from the Pd sample. Finally, 90,202 non-redundant All-unigenes were obtained by splicing and reducing the redundancy of the unigene sequences, with average length and N50 values of 721 nt and 1093 nt, respectively (Table 1). Of the total unigenes, only 814 from Pl and 823 from Pd were longer than 3 kb. The length distributions of the unigenes are shown in S1A Fig. These results showed that the throughput and sequencing quality was high enough for the following analyses.

**Table 1:** Summary of the sequence assembly after Illumina sequencing.

	Samples	Total number	Total length (nt)	Mean length (nt)	N50	Q20 (%)	GC (%)
Read	Pl	48,401,848	#########	90	-	98.31	45.47
	Pd	51,300,426	#########	90	-	98.31	45.69
Contig	Pl	149,470	47,741,711	319	542	-	-
	Pd	144,205	46,839,327	325	579	-	-
Unigene	Pl	88,330	53,279,410	603	989	-	-
	Pd	82,720	50,846,368	615	1018	-	-
	All	90,202	65,006,636	721	1093	-	-

The all-unigene sequences were aligned against protein databases using Blastx (e-value < 0.00001) in the following order: the non-redundant protein database (NR), the Swiss-Prot protein database (Swiss-Prot), the Kyoto Encyclopedia of Genes and Genomes database (KEGG), the Cluster of Orthologous Groups of proteins database (COG), the Gene Ontology database (GO) and the non-redundant nucleotide database (NT) [36]; a total of 44,811 (49.68%) were annotated (S1 Table). The size distribution of the CDSs and predicted proteins are shown in S1B Fig. Additionally, the CDSs of 4,083 all-unigenes that had no hits in Blastx were predicted by ESTScan and then translated into peptide sequences [37]; their size distribution is shown in S1C Fig.

### Functional annotation

Unigene annotation including COG clusters, GO classification and KEGG pathway annotation provides information on expression and function. In the annotation results obtained by Blastx with e-value < 0.00001, 42,699, 26,496, 24,262 and 15,073 All-unigenes were annotated to the NR, Swiss-Prot, KEGG and COG databases, accounting for 47.3%, 29.4%, 26.9% and 16.7%, respectively (S1 Table). Based on the NR annotations and the e-value distribution, 33.43% of the mapped All-unigenes showed strong homology (< 1 e-100), and 66.57% ranged from 1e-5 to 1e-100 (S2A Fig). Additionally, 25.40% of the All-unigenes had > 80% identity with the NR database and 44.01% had similarity ranging from 60–80% (S2B Fig). The majority of sequences (57.91%) shared the highest homology with *Vitis vinifera*, followed by *Ricinus communis* (11.04%), *Populus trichocarpa* (10.74%), *Glycine max* (4.98%), *Medicago truncatula* (2.35%), and *Arabidopsis thaliana* (1.08%); the remaining 4,747 All-unigenes, which consisted of 11.12% of our unique transcripts, showed less than 0.78% similarity to other species (S2 Table).

When searched against the COG database, 15,073 All-unigenes (17.1%) were annotated and classified into 25 different functional classes (S3 Fig). Among these categories, the cluster for ‘General function prediction’, which contained 4,963 All-unigenes (32.93%), represented the largest group, followed by ‘Transcription’ (2,648, 17.57%), ‘Replication, recombination and repair’ (2,496, 16.56%), ‘Posttranslational modification, protein turnover, chaperones’ (2,151, 14.27%) and ‘Signal transduction mechanisms’ (2,038, 13.52%), while only one All-unigene was assigned to ‘Nuclear structure’ as the smallest group (S3 Fig).

**Fig 1:**
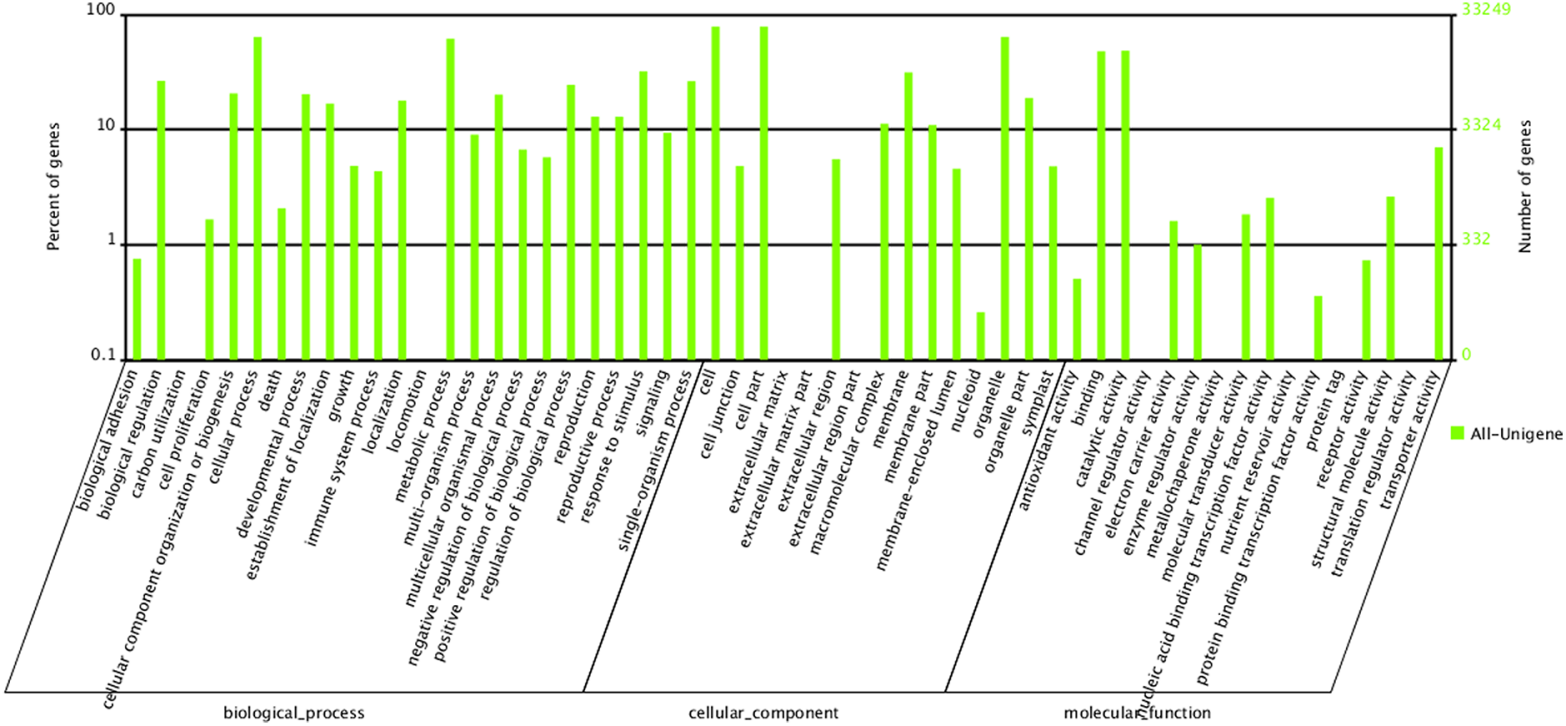
Gene ontology (GO) classification for the *P*. *delavayi* transcriptome. The transcripts (33,249) were categorized into 55 function groups. The right y-axis indicates the number of genes in a category, whereas the left y-axis indicates the percentage of a specific category of genes in the corresponding main category.

In GO analysis, 33,249 All-unigenes were categorized into the three main GO ontologies (molecular function, cellular component and biological process) including 55 functional groups. In the biological process category, the dominant groups were ‘cellular process’ (21,224, 63.83%) and ‘metabolic process’ (20,478, 61.58%). Under the cellular component category, ‘cell’ and ‘cell part’ were the most frequent terms, with the same percentage (26,088, 78.46%). In the molecular function category, ‘binding’ (15,950, 47.97%) and ‘catalytic activity’ (16,117, 48.47%) were predominant, whilst fewer than 10 genes each were categorized into the ‘channel regulator activity’ (7), ‘metallochaperone activity’ (5), ‘protein tag’ (4) and ‘translation regulator activity’ (4) groups (Fig 1).

The KEGG database can help study the complicated biological behaviors of genes and provide pathway annotations [38]. A total of 24,262 All-unigenes were mapped into 128 KEGG pathways, representing metabolism, genetic information processing, organism systems and cellular processes (S3 Table). The two largest pathways were ‘metabolic pathways’ and ‘biosynthesis of secondary metabolites’ with 5,288 and 2,552 All-unigenes, respectively. Two pathways contained only four All-unigenes each, ‘caffeine metabolism’ and ‘betalain biosynthesis’; these were the least represented categories. Many All-unigenes corresponded to flower pigmentation related pathways involved in the biosynthesis of secondary metabolites, including ‘flavonoid biosynthesis’ (179, 0.74%), ‘flavone and flavonol biosynthesis’ (100, 0.41%), ‘anthocyanin biosynthesis’ (10, 0.04%) and ‘isoflavonoid biosynthesis’ (65, 0.27%). These annotations provide a valuable resource for the investigation of specific processes, functions and pathways in *P*. *delavayi*, and the genes in these flavonoid biosynthesis-related pathways might play an important role in flower coloration.

### Transcripts that encode specific genes associated with flower pigmentation

To obtain unique sequences related to flower pigmentation, the non-redundant transcripts associated with anthocyanin biosynthesis were analyzed. As shown in Table 2, we found 66 All-unigenes encoding 11 enzymes involved in the ‘flavonoid biosynthesis’ pathway and potentially related to flower coloration, including 12, 8, 7, 13, 11 and 10 All-unigenes annotated as CHS, CHI, F3H, F3'H, DFR, and FLS, respectively, 2 All-unigenes annotated as flavone synthase II (FNS II), and only 1 All-unigene each aligned to 2'4'6'4-tetrahydroxychalcone 2'-glucosyltransferase (THC2'GT), F3'5'H, and ANS. Additionally, 10 All-unigenes encoding two enzymes involved in the ‘anthocyanin biosynthesis’ pathway, including 3GT and 5GT, were found. Among these All-unigenes, one transcript of each multi-gene enzyme, CHS, CHI, DFR, F3H and ANS, had high sequence similarity to genes isolated from *P*. *suffruticosa*, *PsCHS1* (GenBank: GQ483511), *PsCHI1* (GenBank: GQ984161), *PsDFR1* (GenBank: HQ283448), *PsF3H1* (GenBank: HQ283449) and *PsANS1* (GenBank: HQ283446), respectively [20–22]. This is first time that any of these unigenes have been identified in *P*. *delavayi*.

**Table 2:** Putative unigenes related to flower pigmentation.

Pathway	Gene	Enzyme	Unigene number
Flavonoid biosynthesis	*CHS*	Chalcone synthase	12
	*THC2'GT*	2'4'6'4- tetrahydroxychalcone 2'-glucosyltransferase	1
		Chalcone isomerase	
	*CHI*	Flavanone 3-hydroxylase	8
	*F3H*	Flavanone 3’-hydroxylase	7
	*F3'H*	Flavonoid 3',5'-hydroxylase	13
	*F3'5'H*	Flavonol synthase	1
	*FLS*	Flavone synthase	10
	*FNS II*	Dihydroflavonol-4-reductase	2
	*DFR*	Anthocyanidin synthase	11
	*ANS*		1
Anthocyanin biosynthesis	*3GT*	Anthocyanin 3-O-glycosyltransferase	7
	*5GT*	Anthocyanin 5-O-glucosyltransferase	3

### Analysis of genes differentially expressed between the transcriptomes of Pl and Pd

**Fig 2:**
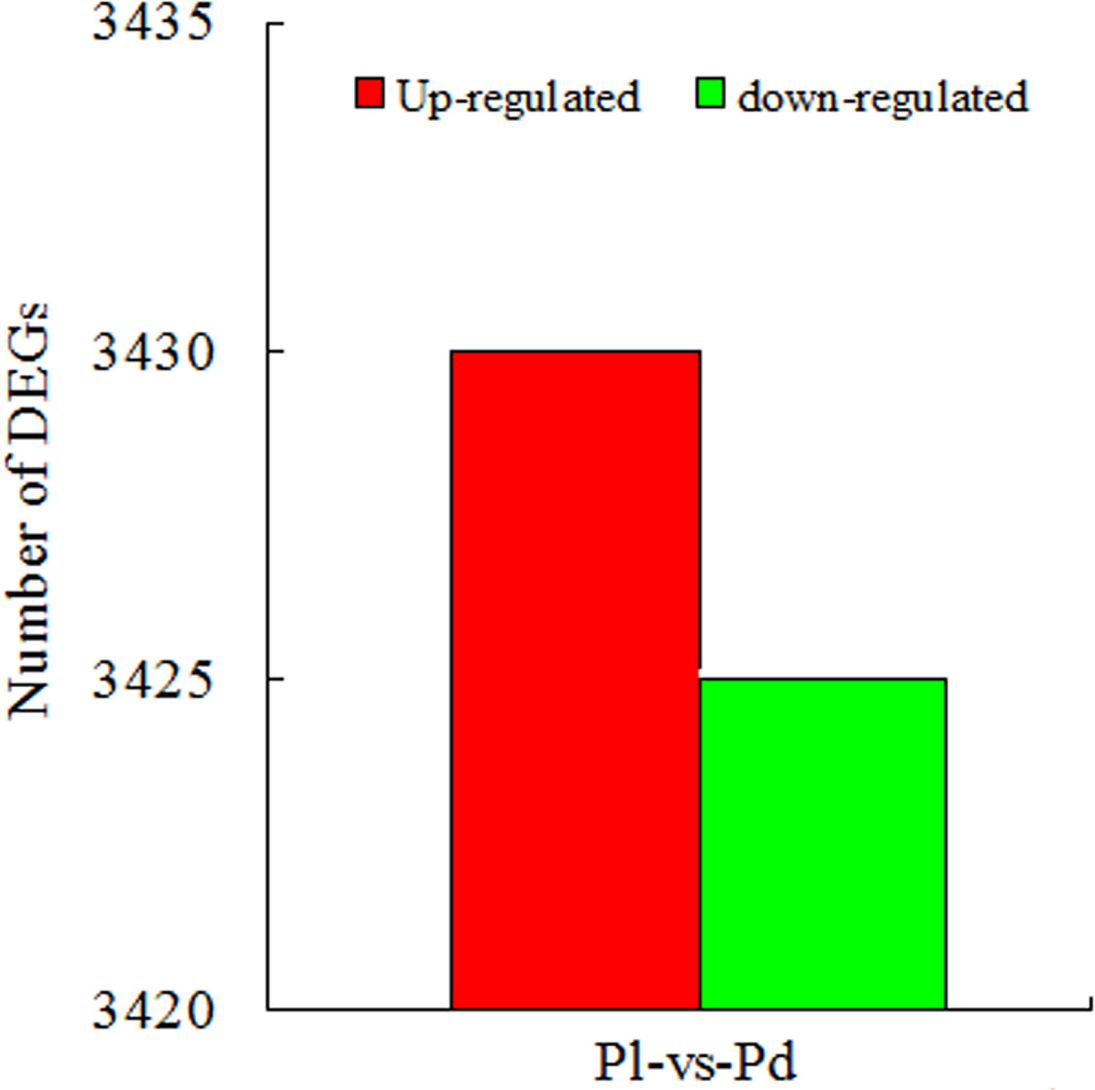
Summary of differentially expressed genes in pairwise comparisons between Pl and Pd.

To identify the candidate genes controlling *P*. *delavayi* yellow and purple-red flower pigmentation, we performed differentially expressed gene (DEG) analysis by calculating fragments per kb per million fragments (FPKM) values [39]. All-unigene differential expression analysis was carried out by comparing the two samples to predict genes with different expression levels. The thresholds false discovery rate (FDR) ≤ 0.001 and absolute value of log_2_ ratio ≥ 1 were used to judge the significance of the DEGs (Pl vs. Pd). A total of 18,784 All-unigenes were identified as differentially expressed, among which 6,855 (36.5%) were aligned with the NR database, with 3,430 up-regulated and 3,425 down-regulated (Fig 2).

### Functional analysis of DEGs

**Fig 3:**
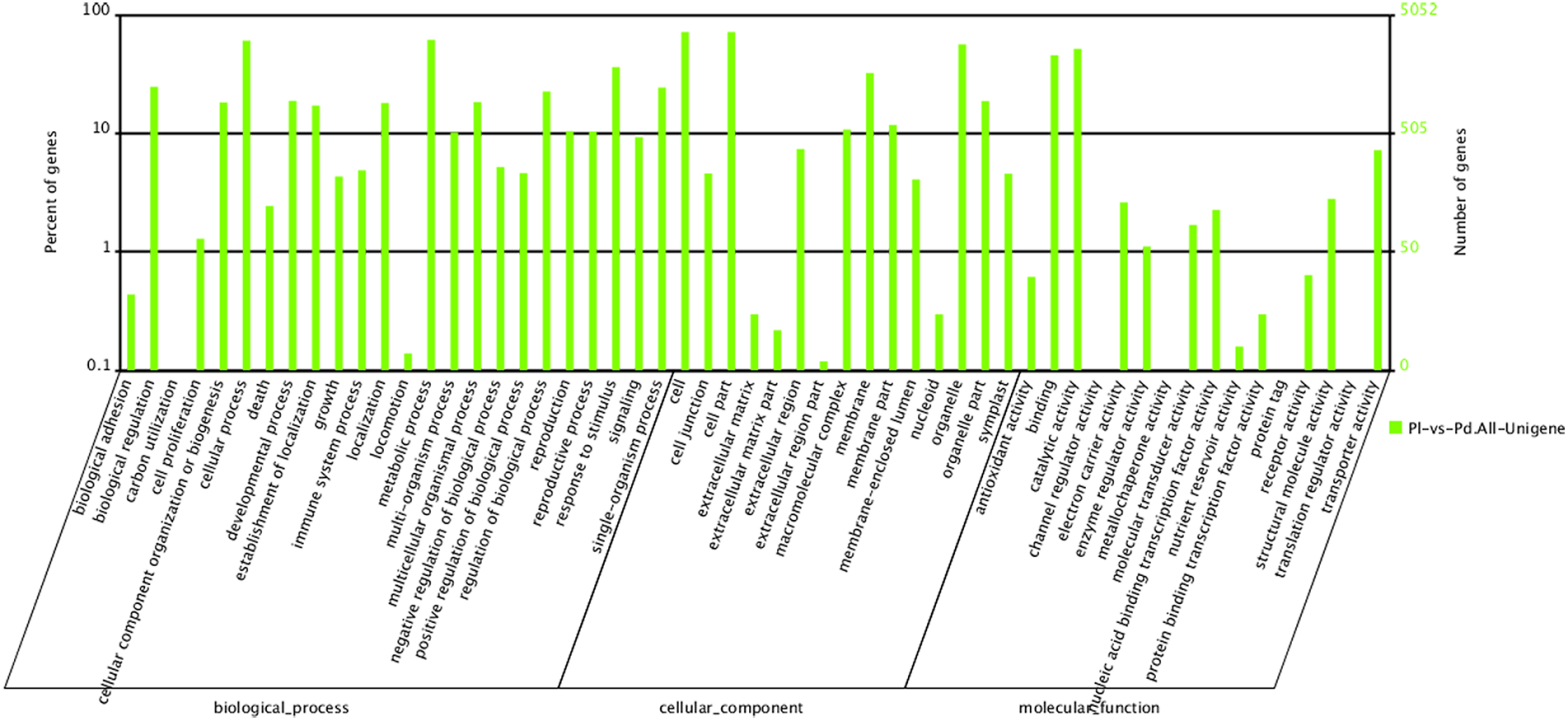
Gene ontology (GO) classification for the DEGs.

GO analysis provided functional classification annotation as well as functional enrichment analysis for the DEGs, and pathway analysis helped to further understand their main biological functions. As shown in Fig 3, 5,052 DEGs were categorized into 55 functional groups, including 24, 15 and 16 groups in the biological process, cellular component and molecular function categories, respectively. In biological process, the major classifications for these All-unigene products were ‘metabolic process’ (3,108, 61.5%) and ‘cellular process’ (3,052, 60.4%). The most frequent cellular component terms were ‘cell’ and ‘cell part’, each with the same number of All-unigenes (3,616, 71.6%). Most of the All-unigenes were classified into the molecular functions ‘catalytic activity’ (2,609, 51.6%) and ‘binding’ (2,303, 45.6%).

**Fig 4:**
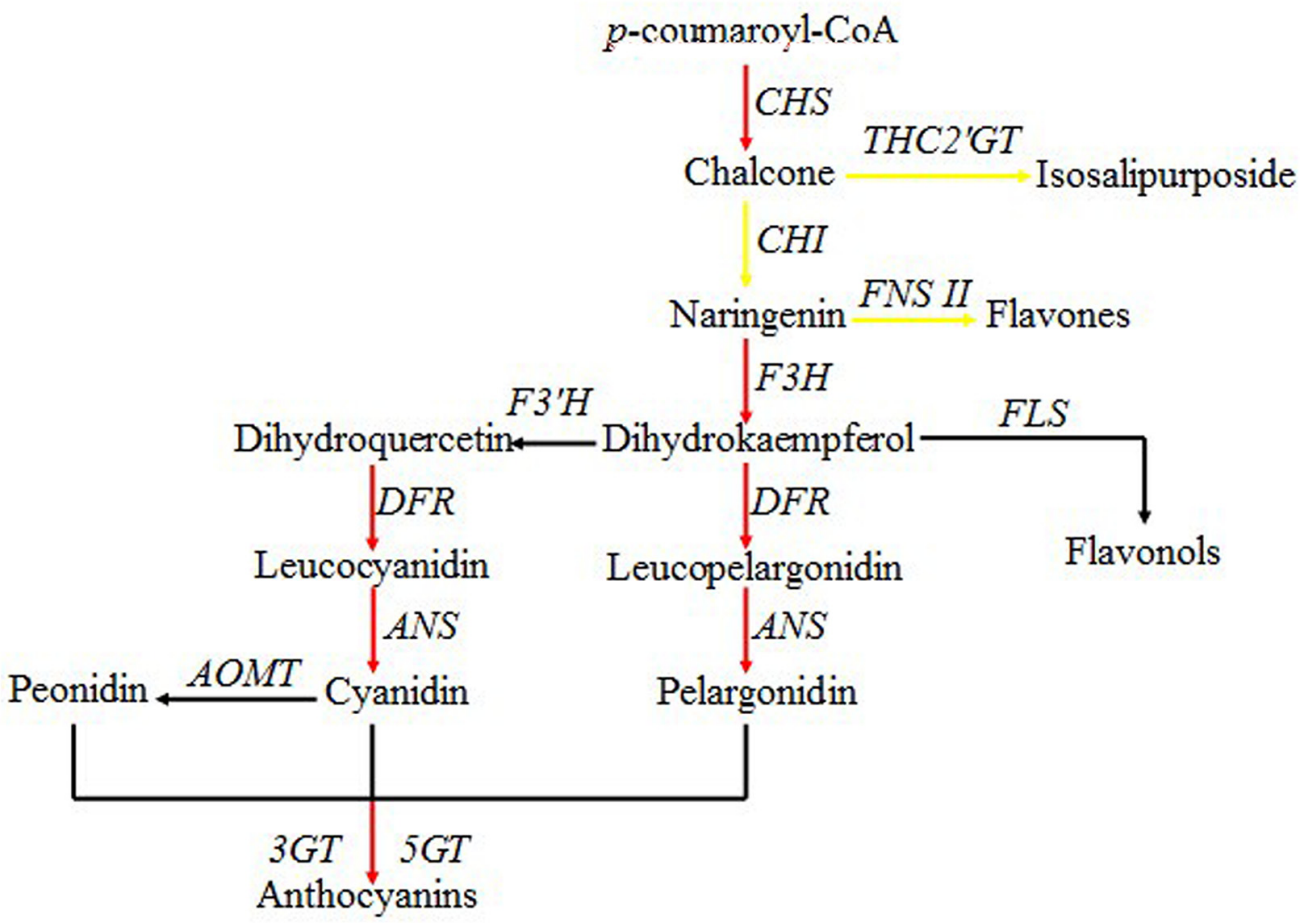
Simplified scheme of the flavonoid biosynthetic pathway in *P*. *delavayi*. Red and yellow lines indicate up-regulated genes in purple-red and yellow petals, respectively.

Many studies have shown that the coloration of yellow and purple-red *P*. *delavayi* petals is closely related to flavonoids [8,12,16]. Using the KEGG database, we mapped 3,969 of 18,784 DEGs to 127 pathways, and noted that 59, 39, and 5 DEGs were involved in ‘flavonoid biosynthesis’ (ko00941), ‘flavone and flavonol biosynthesis’ (ko00944) and ‘anthocyanin biosynthesis’ (ko00942), respectively, and all belonged to the flavonoid biosynthesis pathway. Based on NR, NT and Swiss-Prot annotations, GO functional analysis and KEGG pathway analysis, we subsequently predicted 15 up-regulated and 12 down-regulated All-unigenes involved in petal coloration (Table 3). The annotations for these unigenes included most of the structural genes in the anthocyanin biosynthetic pathway, and the proposed pathway showed that most steps in anthocyanin biosynthesis were up-regulated in purple-red petals (Fig 4).

**Table 3:** Identification of 27 differentially expressed genes involved in *P*. *delavayi* flower pigmentation.

Unigene ID	Pl_FPKM	Pd_FPKM	log2(Pd/Pl)	p-value	FDR	Predicted function
Up-regulated genes (15)						
Unigene7271_All	1.0926	12.414	3.5061	1.41E-60	8.63E-59	CHS
Unigene45464_All	0	3.8864	11.9242	5.11E-07	3.18E-06	CHS
CL7622.Contig1_All	3.2381	10.0341	1.6317	3.16E-15	4.39E-14	CHI
CL2583.Contig1_All	201.7592	820.2684	2.0235	0	0	F3H
CL7561.Contig2_All	5.2308	11.3517	1.1178	4.92E-07	3.09E-06	FLS
CL1376.Contig3_All	0.2903	1.8999	2.7103	1.76E-08	1.34E-07	DFR
CL1376.Contig5_All	0.159	1.189	2.9027	5.71E-05	2.51E-04	DFR
CL1376.Contig6_All	3.0482	226.7622	6.2171	0	0	DFR
Unigene21659_All	1.8912	5.3039	1.4878	1.68E-09	1.43E-08	DFR
Unigene13390_All	62.6394	525.0521	3.0673	0	0	ANS
CL3395.Contig1_All	3.5064	8.4245	1.2646	6.32E-16	9.31E-15	3GT
CL3906.Contig2_All	0.8079	9.6622	3.5801	4.36E-72	3.15E-70	3GT
Unigene11227_All	17.9454	74.0118	2.0441	2.16E-242	5.32E-240	3GT
CL4890.Contig1_All	99.39	242.6426	1.2877	0	0	3GT
CL4890.Contig2_All	110.5854	253.0147	1.1941	0	0	3GT
Down-regulated genes (12)						
CL1498.Contig1_All	9.522	0.696	-3.7741	2.57E-54	1.41E-52	CHS
Unigene18441_All	37.3852	9.5524	-1.9683	5.21E-107	5.57E-105	THC2'GT
CL7622.Contig3_All	39.2223	19.029	-1.0435	5.65E-42	2.33E-40	CHI
Unigene30832_All	11.5703	2.6567	-2.1227	3.52E-08	2.57E-07	CHI
Unigene30833_All	9.5425	1.9625	-2.2817	6.85E-08	4.82E-07	CHI
CL8830.Contig1_All	110.819	0.5231	-7.7269	0	0	FNS II
CL8830.Contig2_All	25.5164	5.8693	-2.1202	2.31E-91	2.13E-89	FNS II
Unigene8606_All	1129.5084	318.1654	-1.8278	0	0	FLS
CL9282.Contig1_All	1.6743	0.3238	-2.3704	3.00E-05	1.39E-04	DFR
Unigene1005_All	8.1856	1.6398	-2.3196	7.78E-09	6.14E-08	3GT
Unigene33533_All	3.1914	0.5424	-2.5568	1.59E-04	6.33E-04	3GT
CL11011.Contig1_All	42.2327	7.2877	-2.5348	1.51E-83	1.26E-81	5GT

Notes: Pl_FPKM, FPKM value in Pl; Pd_FPKM, FPKM value in Pd.

In the flavonoid biosynthetic pathway, the regulation of structural gene expression appears tightly organized in a spatial and temporal way during plant development, and is orchestrated by a ternary complex involving transcription factors from the R2R3-MYB, basic helix–loop–helix (bHLH), and WD40 classes [19]. Thus, besides structural genes, we also investigated the expression levels of these three transcription factor families. As shown in S4 Table, 50 differentially expressed All-unigenes were annotated as transcription factors between yellow and purple-red petals, including 21 All-unigenes encoding R2R3-MYBs, 12 encoding bHLHs and 17 encoding WD40s. Among these transcription factors, 21 were highly expressed in purple-red petals and 29 were highly expressed in yellow petals.

### Expression analysis of candidate DEGs probably involved in flavonoid biosynthesis

**Fig 5:**
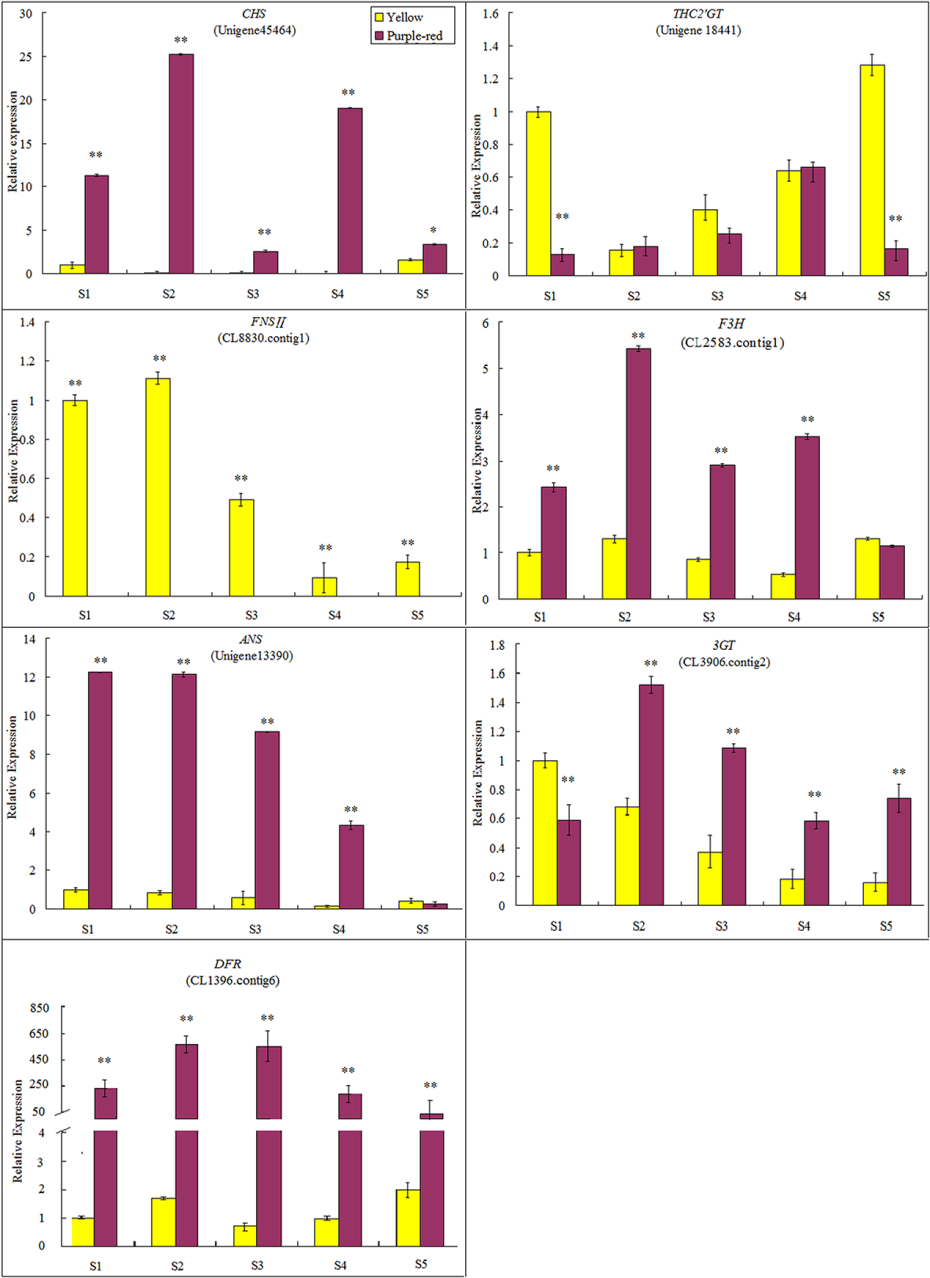
qRT-PCR analysis of seven flavonoid biosynthetic pathway-related candidate unigenes in Pl and Pd. qRT-PCR analysis was performed using total RNA from petals at each floral developmental stage (S1–S5). The expression in yellow petals at stage 1 was used as a calibration standard. Data from real-time PCR were normalized to the *helicase* gene expression value, and relative transcript levels are presented as means with standard errors (S.E.) of five replications.

To validate the unigene expression profiles from the transcriptome sequencing data, seven flavonoid biosynthesis-related All-unigenes that exhibited particularly strong expression in anthocyanin-pigmented petals compared with other flower organs, such as sepals, carpels, leaves, and stamens (data not shown), and showed the largest difference in expression level between purple-red and yellow petals, were selected for quantitative RT-PCR assays. To get more information in this analysis, we examined the expression patterns of these genes at five different developmental stages during flower opening. As shown in Fig 5, purple-red petals showed much higher gene expression levels than yellow petals for most of the selected genes, including *CHS*, *F3H*, *DFR*, *ANS* and *3GT*. Among them, *DFR* in particular was expressed at a statistically significantly higher level in purple-red petals than in yellow ones all through the flower pigmentation period, with *p* < 0.01 (Fig 5), suggesting that it might play an extremely important role in purple-red petal coloration.

Conversely, the expression levels of the other two selected genes were much higher in yellow petals (Fig 5). *THC2ʹGT* showed prominent expression at stage 1 prior to petal pigmentation and at stage 5 when the petals were fully pigmented, both with almost 8-fold greater expression than in purple-red petals at the corresponding stages, implying that a high expression level of this gene prior to petal pigmentation is essential for yellow pigment accumulation. For *FNS II*, the transcript levels were extremely high in yellow petals but hardly detected in purple-red ones throughout the entire process of flower pigmentation. These results demonstrated that the expression patterns of the seven selected DEGs analyzed by real-time RT-PCR were consistent with their respective digital expression data.

## Discussion

In recent years, as a cost-effective and efficient tool to discover novel genes and analyze molecular mechanisms, high-throughput mRNA sequencing based on the Illumina/Solexa platform has been widely used for transcriptome analysis of many ornamental plants without genomic sequence data, such as *P*. *suffruticosa* [33,34], *Gardenia jasminoides* [40], *Chrysanthemum lavandulifolium* [41] and *Zantedeschia aethiopica* [42].

Prior to this study, most sequencing efforts in wild tree peony species were based on EST sequencing; very few tags had been reported in public databases and little genetic or genomic information was available. Here, we report the results of a large-scale transcriptome analysis of petal pigmentation in an endemic species of China, *P*. *delavayi*, using RNA-Seq technology. Two cDNA libraries constructed from the petals of yellow (Pl) and purple-red (Pd) flowers at different opening stages were analyzed, leading to the identification of 44,811 unigenes that were annotated using known sequences in various databases and DEGs involved in flavonoid biosynthesis. The relative transcript levels of seven candidate DEGs were further validated using real-time PCR. To the best of our knowledge, this is the first attempt to use Illumina paired-end sequencing technology for the *de novo* sequencing and assembly of the *P*. *delavayi* flower transcriptome. We believe our data will provide important new insights and facilitate further studies of *P*. *delavayi* genes and their functions.

Compared with previous floral transcriptome studies in the tree peony *P*. *suffruticosa* [33, 34] and other woody ornamental plants, such as *Rosa* [43], *Camellia chekiangoleosa* [41] and *G*. *jasminoides* [40], we herein report more contigs and unigenes, suggesting that the wild tree peony species *P*. *delavayi* contains abundant gene resources. In previous studies of peach [44] and safflower [27] flowers, transcriptome dynamics associated with petal pigmentation were investigated. Millions of transcripts were generated from petals of different colors, and enriched functional category analysis based on GO annotations showed that genes associated with ‘cell’, ‘cell part’, ‘binding’, ‘cellular processes’, ‘metabolic processes’ and ‘catalytic activity’ were the highest-represented groups. In the present study, similar results were obtained (Fig 1), indicating that the *P*. *delavayi* flowers were undergoing rapid development and carrying out intensive metabolic activities with complicated regulation by many transcription factors during petal pigmentation.

The accumulation of secondary metabolites plays an important role in the formation and development of flower color, especially flavonoids [45,46]. Flavonoid biosynthesis is regulated by a series of structural and regulatory genes, and the structural genes directly control flavonoid biosynthesis and accumulation [47]. A number of researchers have demonstrated that the primary pigments related to *P*. *delavayi* flower color are flavonoid compounds, including chalcones, flavones and flavonols, and anthocyanins, mainly Pn3G5G, which contribute to a variety of colors, such as yellow, orange, and purple-red [8,12,13,16]. The flower coloration of yellow *P*. *delavayi* individuals is mainly due to the biosynthesis of the 2’-glucoside of 2’,4’,6’,4-tetrapydroxychalcone (THC)-isosalipurposide (ISP), chrysoeriol and apigenin [8,16], whereas purple-red coloration is dependent on anthocyanin contents, particularly peonidin-3,5-glucosides (Pn3G5G) [12]. Therefore, in this work, unigenes participating in the flavonoid biosynthetic pathway were specifically selected and studied in detail, providing new insight into the regulation of flavonoid and anthocyanin biosynthesis in *P*. *delavayi* that should accelerate engineering of this pathway in the future.

As shown in Table 2, almost all of the structural genes in the main flavonoid biosynthesis pathway were identified, indicating that this pathway is well conserved in *P*. *delavayi*. We also observed that many of these genes appeared to form multi-gene families, implying that the genome of *P*. *delavayi*, like those of many other higher plants, has undergone one or more rounds of genome duplication during its evolution. Based on the results of unigene differential expression analysis, we predicted 27 DEGs as key structural genes involved in coloration from our sequencing data, including 15 up-regulated and 12 down-regulated genes (Table 3). In this pathway (Fig 4), CHS is the first committed enzyme, catalyzing the condensation of one molecule of 4-coumaroyl-CoA and three molecules of malonyl-CoA to generate 4,2′,4′,6′-tetrahydroxychalcone. We analyzed the expression patterns of 12 novel transcripts related to *CHS* and found that most of them, especially Unigene45464_All, were significantly up-regulated in purple-red petals (Fig 5). We propose that a sharp increase of transcripts encoding CHS may provide more substrate for anthocyanin biosynthesis, which is mainly responsible for petal coloration in purple-red flowers. 4,2′,4′,6′-Tetrahydroxychalcone is a key intermediate in *P*. *delavayi* flower coloration; it can be glycosylated by *THC2′GT* or isomerized by *CHI*, leading to the production of chalcone isosalipurposide and other flavonoids, such as flavones and flavonols, and anthocyanins, which contribute to the yellow and purple-red colors seen in *P*. *delavayi*, respectively. Mutations of the *CHI* gene have been shown to be necessary for isosalipurposide accumulation in carnation [48] and barley [49]. Additional mutation of the *DFR* gene confers better yellow coloration in carnation [50]. The existence of several genes encoding THC2′GT in carnation [51, 52] indicates that several non-specific enzymes may catalyze the reaction *in vivo*. In this work, only Unigene18441_All showed sequence homology to *THC2'GT* from *Catharanthus roseus* (GenBank: BAF75901) [53], and its expression in yellow petals was almost 2-fold higher than in purple-red petals, suggesting an important role in yellow flower pigmentation. Interestingly, among the eight novel transcripts related to *CHI*, only one unigene (CL7622.Contig1_All) was down-regulated in yellow petals. Additionally, both of the transcripts (CL8830.Contig1_All and CL8830.Contig2_All) annotated as *FNS II*, which is responsible for flavone formation [54, 55], were obviously up-regulated in yellow petals. Park et al. [56] reported that in *Scutellaria baicalensis*, *SbCHI*-overexpressing hairy root lines not only showed enhanced *SbCHI* gene expression, but also produced more flavones than the control. Thus, considering that *P*. *delavayi* yellow flowers are pigmented by the yellow chalcone isosalipurposide and by colorless or yellow flavonol and flavone glycosides, we speculate that the high co-expression of *THC2'GT*, *CHI* and *FNS II* ensures the accumulation of pigments contributing to the yellow color.

Regarding the downstream enzymatic genes involved in the *P*. *delavayi* anthocyanin biosynthetic pathway, *F3H*, *DFR*, *ANS* and *3GT* were expressed at a significantly higher level in purple-red petals than in yellow petals according to both transcriptome profiling and qRT-PCR analysis (Table 3, Fig 5). We can see from Fig 4 that F3H catalyzes the formation of DHK, which can be further hydroxylated by F3'H to form DHQ. Then, DHK and DHQ are deoxidized to leucoanthocyanidins by DFR, leading to synthetic branches catalyzed by ANS and GT that produce corresponding pelargonidin-based (orange to red) and cyanidin-based (red to magenta) pigments, respectively. Cyanidin is the precursor pigment of other anthocyanidins, and can be transformed into peonidin derivatives by the action of a 3′-O-methyltransferase [57–59]. Because the purple-red color in *P*. *delavayi* flowers is mainly related to the synthesis of cyanidin-derived anthocyanins (cyanidin and peonidin based), our results suggest that high expression levels of *F3H*, *DFR*, *ANS* and *3GT* genes induce cyanidin and peonidin production to make flowers purple-red. In contrast, the low transcription levels of these genes observed in yellow petals ensure the inhibition of anthocyanin formation. Thus, we propose that the molecular mechanism underlying the differences in *P*. *delavayi* flower pigmentation is related primarily to structural genes downstream of *CHI* in the flavonoid synthetic pathway. These results are consistent with previous studies on flower color in many other plants [60–62] and the petal pigments of yellow and purple-red *P*. *delavayi* individuals [8,12,16].

Transcriptional regulation of structural genes appears to be a major mechanism by which anthocyanin biosynthesis is regulated in plants. R2R3-MYB, bHLH and WD40 proteins represent the three classes of transcription factors influencing anthocyanin biosynthesis intensity and patterns, and generally controlling the expression of many different structural genes [63–65]. A large number of previous reports have demonstrated that transcription factors, especially R2R3-MYBs, play an important role in color differences in many crops, including grape [66], apple [67], cauliflower [68], oriental hybrid lily [69], and flowering peach [70]. In this study, we detected 50 differentially expressed transcription factors from these three classes, and found that some were significantly up-regulated in purple-red petals and showed positive correlation with *F3H*, *DFR*, *ANS* and *3GT* genes in the anthocyanin biosynthesis pathway (S4 Table). These results provide an informative list of candidate regulatory transcription factors involved in *P*. *delavayi* flower pigmentation, but further research related to these candidate regulatory genes including sequence, expression and biological function analyses needs to be carried out.

## Conclusions

The Illumina HiSeq sequencing technology was used for sequencing and transcriptome analysis of the non-model plant *P*. *delavayi*, and provided an efficient approach for identifying critical genes involved in *P*. *delavayi* flower pigmentation. We identified potential candidate genes encoding key enzymes and reconstructed the flavonoid biosynthetic pathway in *P*. *delavayi*. The up-regulated unigenes encoding *F3H*, *DFR*, *ANS* and *3GT* might play an important role in purple-red petal pigmentation, while high co-expression of *THC2'GT*, *CHI* and *FNS II* ensures the accumulation of pigments contributing to the yellow color. In addition, differentially expressed transcription factors related to anthocyanin biosynthesis were detected. Our results will provide valuable resources and a substantial basis for understanding the molecular mechanisms controlling *P*. *delavayi* flower pigmentation, and will eventually accelerate the genetic engineering of flower color in tree peony.

## Methods

### Ethics Statement

All Plant protocols were reviewed and approved by the Research Institute of Forestry, Chinese Academy of Forestry, State Forestry Administration. All necessary permits were obtained for field studies from the Diqing Forestry Bureau, Shangri-La County, Yunnan Province, China. The fieldwork conducted for sampling did not affect the local ecology and did not involve endangered species.

### Plant materials

**Fig 6:**
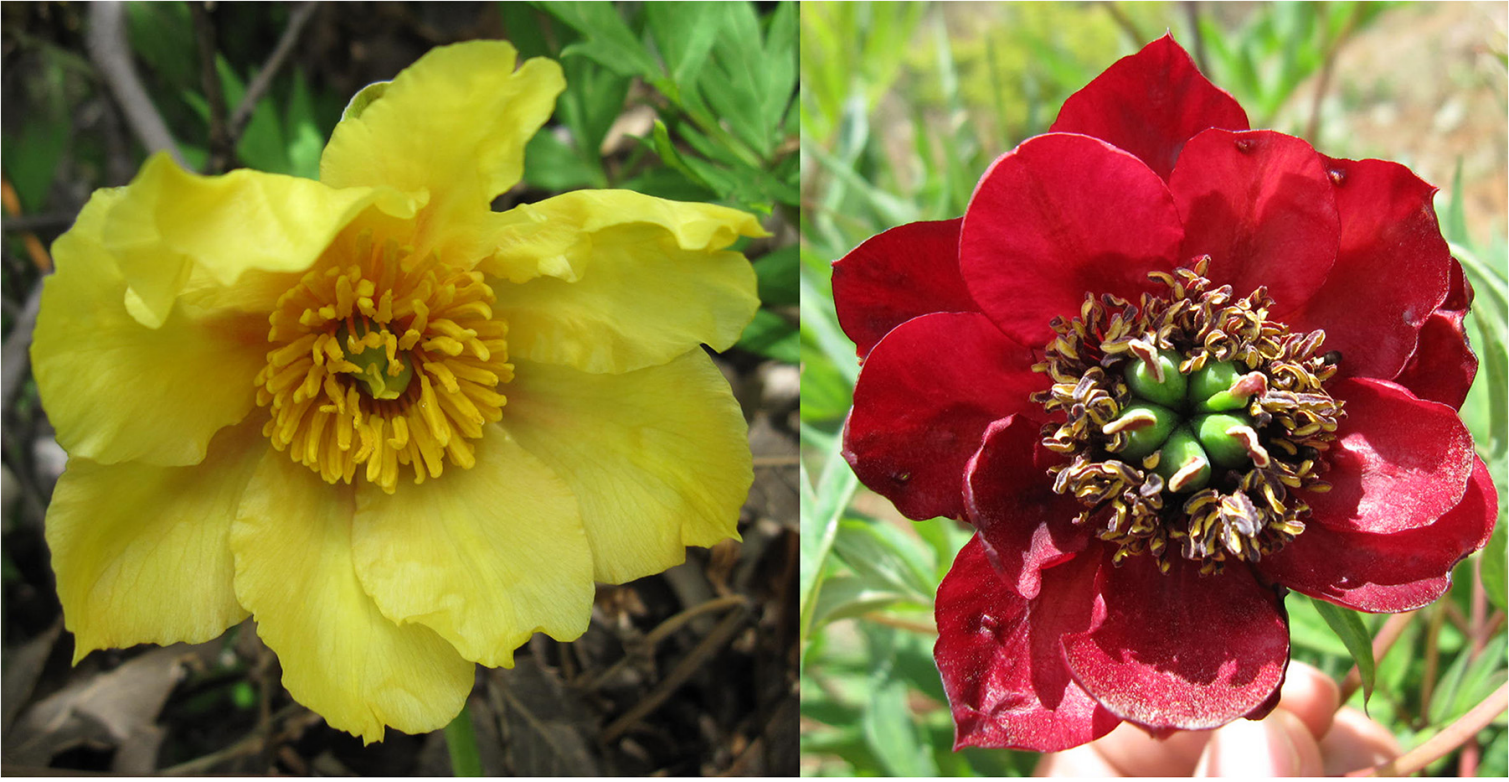
Fully open flowers of individuals selected for sequencing. A, yellow flowered individual; B, purple-red flowered individual.

Petal samples were separately detached from purple-red-flowered and yellow-flowered individuals from a wild *P*. *delavayi* population in Shangri-La County (27°57′N, 99°35′E), Yunnan Province, China (Fig 6) at five different opening stages in the afternoon during the end of April to early May, 2013. The flower opening stages were described by Zhou et al. [20]: stage 1, unpigmented tight bud; stage 2, slightly pigmented soft bud; stage 3, initially opened flower; stage 4, half opened flower; stage 5, fully opened and pigmented flower with exposed anthers. The petals were immediately frozen in liquid nitrogen and after transport to the laboratory stored at −80°C until RNA extraction.

### RNA extraction, cDNA library construction and transcriptome sequencing

Total RNA was extracted from petals by the cetyltrimethylammonium bromide (CTAB) method with some modification [71]. RNase-free DNase I (Tiangen; Beijing, China) was applied to remove contaminating genomic DNA. The RNA purity was determined with a NanoDrop 2000 spectrophotometer (NanoDrop Technologies; Wilmington, DE, USA) and 2% agarose gels were run to verify RNA integrity. Two cDNA libraries were constructed by mixing equal amounts of RNA from purple-red or yellow petals at five different opening stages, and named Pd and Pl, respectively. Enrichment of mRNA, fragment interruption, addition of adapters, size selection and PCR amplification and RNA-Seq were performed by staff at the Beijng Genome Institute (BGI; Shenzhen, China). mRNA was isolated with magnetic oligo (dT) beads. The mRNA was mixed with fragmentation buffer, fragmented into short fragments and used as a template for first-strand and second-strand cDNA synthesis. The cDNA fragments were purified using a QiaQuick PCR extraction kit (Qiagen; Valencia, CA, USA), and resolved with elution buffer for end reparation and single nucleotide A (adenine) addition. The short fragments were connected with adapters and suitable fragments were selected for PCR amplification as templates. An Agilent 2100 Bioanalyzer (Agilent Technologies; Palo Alto, CA, USA) and an ABI StepOnePlus Real-Time PCR System (Applied Biosystems; Foster City, CA, USA) were used during the quality control steps. Finally, the two libraries were sequenced at the BGI using an Illumina HiSeq 2000 sequencing platform (Illumina Inc.; San Diego, CA, USA).

### *De novo* assembly and sequence analysis

The raw reads were first filtered by removing adaptor sequences, empty reads, repeated reads and low-quality reads to get clean reads for subsequent analysis. The sequencing data for the clean reads were deposited in the National Center for Biotechnology Information (NCBI) Sequence Read Archive (http://www.ncbi.nlm.nih.gov/Traces/sra) with accession number SRP052291. Then, *de novo* assembly of the clean reads was performed to generate non-redundant unigenes using the Trinity software with an optimized *k*-mer length of 25 [35].

For sequence analysis, the resulting unigenes were aligned by Blastx to several protein databases (e-value < 0.00001) such as NCBI NR, Swiss-Prot, KEGG and COG, and the best-aligned results were used to decide the sequence direction of the unigenes. If the results from different databases conflicted, a priority order of NR > Swiss-Prot > KEGG > COG was followed when deciding the sequence direction of the unigenes. If a unigene was unaligned to any of the above databases, the ESTScan software was used to decide its sequence direction [37]. For unigenes with sequence directions, we recorded their sequences from the 5′ end to the 3′ end; for those without any direction, the sequences were provided by the assembly software. The length of the sequences assembled was a criterion of assembly success. The contig and unigene length distributions were calculated.

### Functional annotation of unigenes

To obtain functional annotations, unigene sequence-based alignments were performed against three public databases (NR, Swiss-Prot and KEGG; e-value < 0.00001), and domain-based alignments were carried out against the COG database (e-value < 0.00001). Additionally, a Blastn search was performed against the NCBI NT database with an e-value < 0.00001. The proteins with the highest sequence similarity to the unigenes were retrieved for analysis. KEGG mapping was used to study the complex biological behavior and obtain pathway annotations for the unigenes, while COG matched each annotated sequence to an ancient conserved domain, and was used to predict and classify the possible functions of the unigenes. Based on NR annotations, the Blast2GO program (version 2.3.5, http://www.blast2go.de/) was used to retrieve associated GO terms describing biological processes, molecular functions and cellular components [72]. After getting GO annotations, the WEGO (http://wego.genomics.org.cn/cgi-bin/wego/index.pl) software was used to perform GO functional classification for all unigenes and to understand the distribution of gene functions of the species at the macro level [73].

### Gene expression analysis using RNA-Seq data

Unigene expression was calculated using the FPKM method [39], which eliminates the influence of different gene lengths and sequencing level on the calculation of gene expression. The false discovery rate (FDR) control is a statistical method used in multiple hypothesis testing to correct for *p*-value. When we obtained the FDR, we used the ratio of FPKMs from the two samples at the same time. The smaller the FDR and the larger the ratio, the larger the difference in expression level between the two samples; therefore, for this analysis, thresholds of FDR ≤ 0.001 and ratio > 2 were chosen to judge the DEGs (Pl vs. Pd) [74]. The confirmed DEGs were subjected to GO functional enrichment analysis and KEGG pathway analysis. Then, based on NR, NT and Swiss-Prot annotations, GO functional analysis, KEGG pathway analysis and flower coloration studies in tree peony [8,11–15,20–22], the DEGs involved in petal coloration were screened for up/down-regulated unigenes, among which those DEGs with the highest absolute values for the FPKM ratio (Pl vs. Pd) were predicted as key genes for petal coloration and used for further analysis.

### Quantitative Real-time PCR (qRT-PCR) Analysis

qRT-PCR was performed to verify the expression of seven genes probably involved in purple-red and yellow flower pigmentation in *P delavayi*. Total RNA was extracted from petals at five different opening stages. After treatment with RNase-free DNase I (Tiangen; Beijing, China) according to the user manual, 1 μg of total RNA was reverse-transcribed to first-strand cDNA using the PrimeScript RT reagent kit (Takara; Otsu, Japan). qRT-PCR experiments were performed in a 96-well plate with an ABI Prism 7500 Sequence Detector (Applied Biosystems, USA), using a SYBR Premix Ex TaqKit (Takara; Otsu, Japan) to monitor cDNA amplification, according to the manufacturer’s protocol. As a control, parallel amplification reactions for the tree peony housekeeping gene *helicase* (GenBank: EF608942) were also performed. Each primer set was designed based on the 3′-end cDNA sequence of the corresponding gene using the Primer Premier 5.0 software; all primer sequences are listed in S5 Table. Each 20 μL PCR reaction contained 10 μL of SYBR Premix Ex Taq (2×), 2 μL of 20× diluted RT-product, 0.8 μL of each forward and reverse primers (10 μM), 0.4 μL of ROX Reference Dye II, and 6 μL of ddH_2_O. The thermal conditions were 95°C for 30 s and 40 cycles of 95°C for 5 s and 60°C for 35 s, and then 95°C for 15 s, 60°C for 20 s and 95°C for 15 s for the dissociation stage. After the real-time PCR, the absence of unwanted by-products was confirmed by automated melting curve analysis and agarose gel electrophoresis of the PCR products. In all experiments, five replicates for each RNA sample were included; averages were calculated and differences in the threshold cycle (*C*t) were evaluated by the 7500 System Sequence Detection Software v1.3.1.

For data analysis, the relative expression ratios were calculated by the comparative ΔΔ*C*t method (ABI Prism 7500 Sequence Detection System, Applied Biosystems, USA) of relative gene quantification. To monitor the expression patterns of the seven selected transcripts during flower pigmentation, the relative quantification of gene expression was achieved by calibrating the transcription level in petals at different stages to that in yellow petals at stage 1. The expression level calculated by the formula 2^−ΔΔCt^ represents the *x*-fold difference from the calibrator.

## Supporting Information

S1 Fig Overview of Pl and Pd transcriptome assembly. A, Length distribution of the unigenes obtained from our *de novo* assembly of contigs; B, length distribution of the CDSs produced by searching unigene sequences against various protein databases and proteins predicted from the CDSs; C, length distribution of ESTs obtained from the ESTScan results.
(TIF)

S2 Fig Characteristics of the homology search of the unigenes. E-value (A) and similarity (B) distributions of the top Blastx hits against the NR database for each unigene.
(TIF)

S3 Fig Classification of the clusters of orthologous groups (COG) for the *P*. *delavayi* transcriptome. The unigenes (15,073) were annotated and divided into 25 specific categories.
(TIF)

S1 Table Statistics for the annotation results. (XLS)

S2 Table Species distribution of the top Blastx hits in the NR database. (XLS)

S3 Table Metabolic pathway analysis of the *P*. *delavayi* unigenes conducted using the Kyoto Encyclopedia of Genes and Genomes (KEGG) annotation system. (XLS)

S4 Table Three transcription factors families differentially expressed between yellow and purple-red petals. (XLS)

S5 Table Gene names, sequences and all primers used for qRT-PCR analysis. (XLS)
