# Supplementary figures and images for: Transcriptomic Analysis of Paeonia delavayi Wild Population Flowers to Identify Differentially Expressed Genes Involved in Purple-Red and Yellow Petal Pigmentation
Source: PLoS One. 2015 Aug 12;10(8):e0135038. doi: 10.1371/journal.pone.0135038 (PMC4534100; doi:10.1371/journal.pone.0135038)

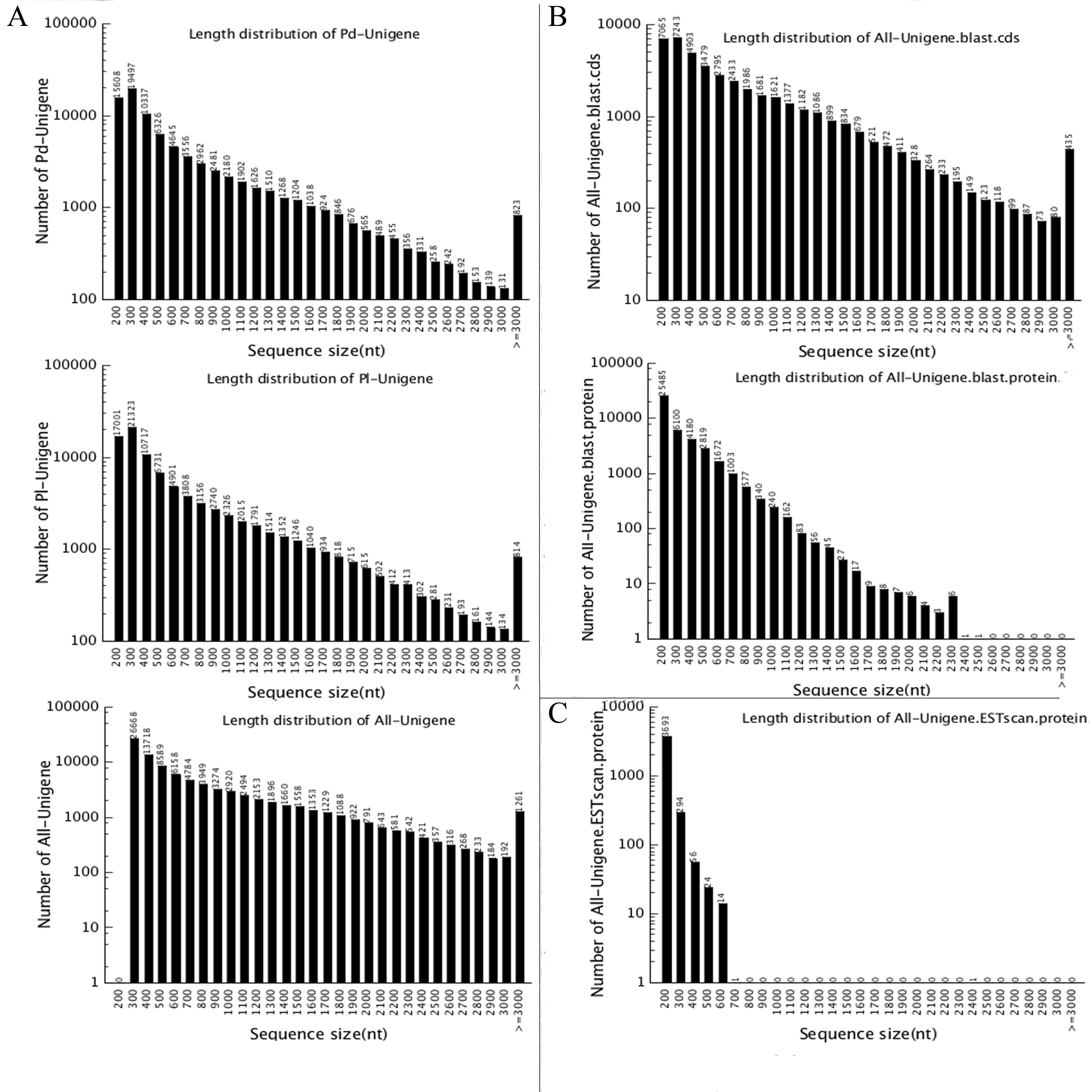

Supplement: S1 Fig — A, Length distribution of the unigenes obtained from our de novo assembly of contigs; B, length distribution of the CDSs produced by searching unigene sequences against various protein databases and proteins predicted from the CDSs; C, length distribution of ESTs obtained from the ESTScan results. (TIF) [file pone.0135038.s001.tif]

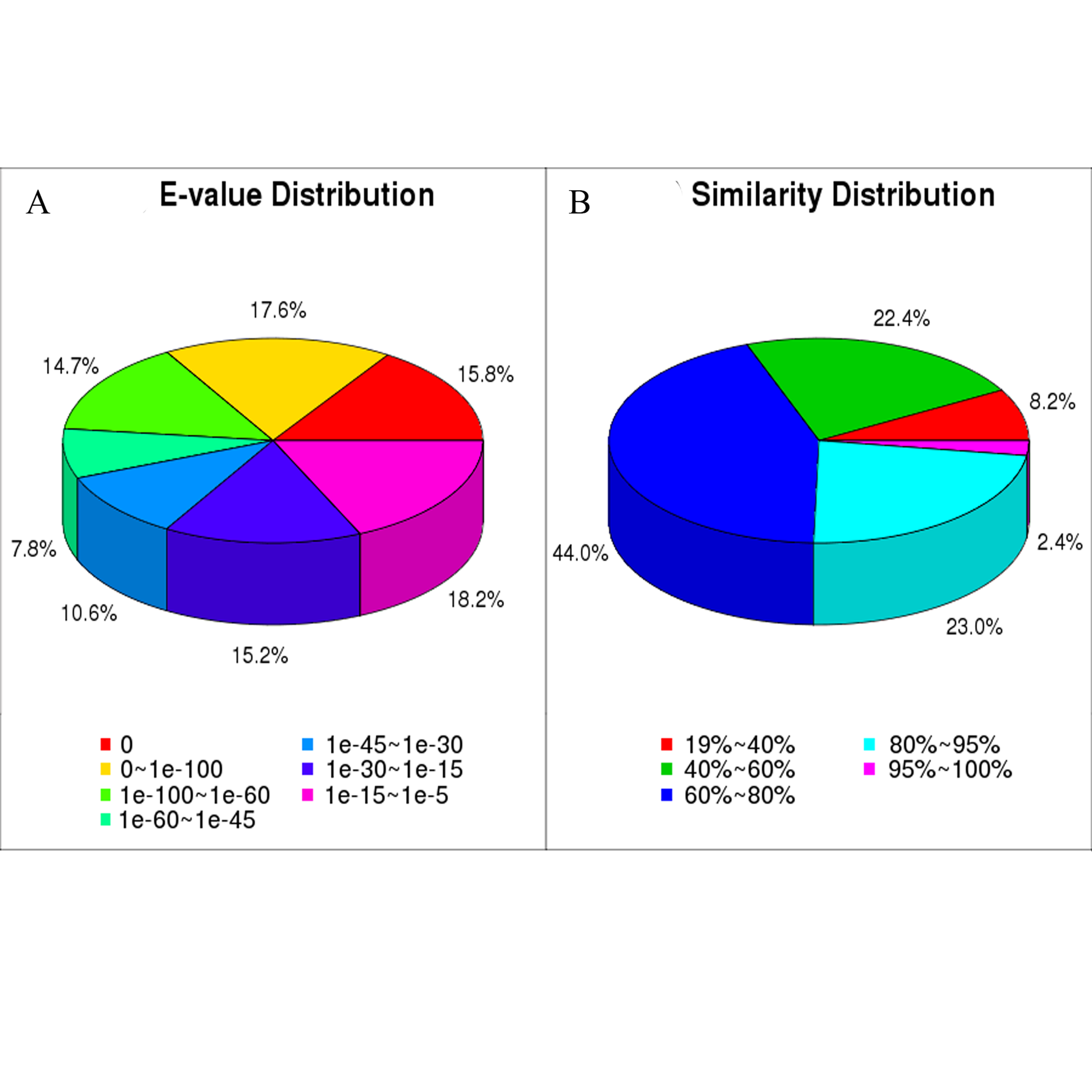

Supplement: S2 Fig — E-value (A) and similarity (B) distributions of the top Blastx hits against the NR database for each unigene. (TIF) [file pone.0135038.s002.tif]

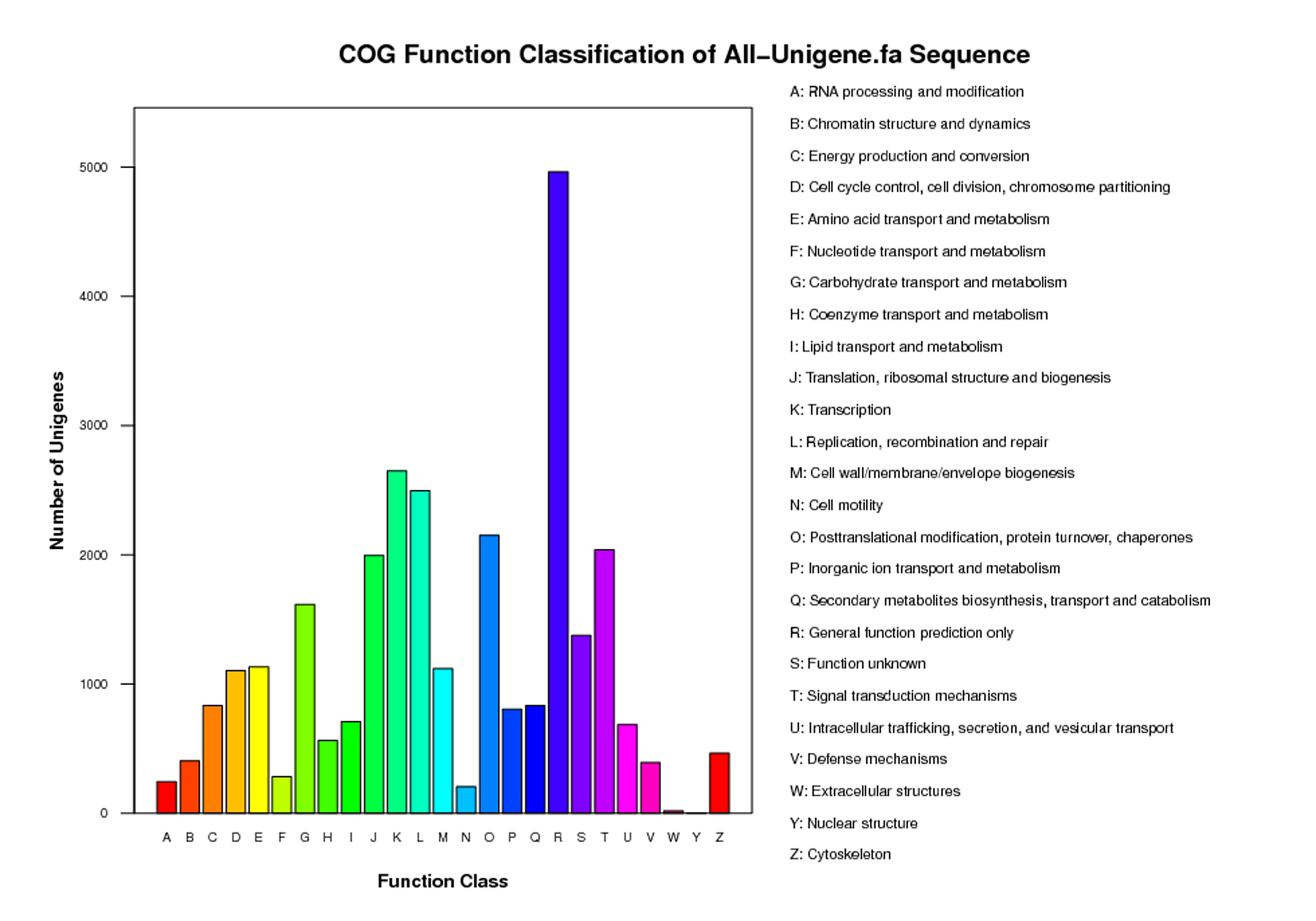

Supplement: S3 Fig — The unigenes (15,073) were annotated and divided into 25 specific categories. (TIF) [file pone.0135038.s003.tif]
